# Lysophosphatidylcholine-DHA Specifically Induces Cytotoxic Effects of the MDA-MB-231 Human Breast Cancer Cell Line *In Vitro*—Comparative Effects with Other Lipids Containing DHA

**DOI:** 10.3390/nu15092137

**Published:** 2023-04-29

**Authors:** Dalal Mohamad Ali, Kevin Hogeveen, Rose-Marie Orhant, Tiphaine Le Gal de Kerangal, Françoise Ergan, Lionel Ulmann, Gaëlle Pencreac’h

**Affiliations:** 1 BiOSSE: Biology of Organisms, Stress, Health, Environment, IUT de Laval, Département Génie Biologique, Le Mans Université, F-53020 Laval, France; 2 Toulouse Biotechnology Institute, Equipe CIMEs, Université de Toulouse, CNRS, INRAE, INSA, F-31077 Toulouse, France; 3 Unité de Toxicologie des Contaminants, ANSES, F-35306 Fougères, France

**Keywords:** lysophosphatidylcholine-DHA, human breast cancer, MDA-MB-231, high content analysis, oxidative stress, membrane damage

## Abstract

Docosahexaenoic acid (DHA, C22:6 ω-3) is a dietary polyunsaturated fatty acid that has an important role in human health. Epidemiological studies linked a high intake of DHA to a reduced risk of certain cancers. Recently, attention focused on how the lipid carrier in which DHA is delivered, i.e., esterified on acylglycerols, phospholipids, or free, affects its biological effects. However, studies comparing the effects of these different forms for DHA supply to cancer cells in vitro are limited. In this study, the effect of free DHA and five lipids carrying one to three DHA chains (LPC-DHA, PC-DHA, MAG-DHA, DAG-DHA and TAG-DHA) on the viability of the MDA-MB-231 breast cancer cell line was compared. Our results revealed a strong structure–function relationship of DHA-carrying lipids on the viability of MDA-MB-231 cells. Glycerophosphocholine-based lipids are the most effective DHA carriers in reducing the viability of MDA-MB-231 cells, with LPC-DHA being more effective (IC_50_ = 23.7 µM) than PC-DHA (IC_50_ = 67 µM). The other tested lipids are less toxic (MAG-DHA, free DHA) or even not toxic (DAG-DHA, TAG-DHA) under our conditions. Investigating the mechanism of cell death induced by LPC-DHA revealed increased oxidative stress and membrane cell damage.

## 1. Introduction

DHA (all *cis* 4, 7, 10, 13, 16, 19- docosahexaenoic acid, C22:6 ω-3) is a polyunsaturated fatty acid (PUFA) of marine origin, that belongs to the ω-3 family (ω-3 PUFA). DHA is known to have beneficial effects on the brain and was linked to healthy aging throughout life. It also has a role in the prevention of eye disease and heart disease [1,2,3].

Marine microalgae are one of the dominant producers of DHA in the biosphere and are a major source of DHA for fish and other marine animals, which, in turn, are dietary sources of DHA for humans [3,4]. Thus, DHA is transferred from microalgae to humans along the food chain, mainly through the lipids of fatty fish such as salmon [5]. Throughout the food chain, DHA is mostly found in esterified form in complex lipids, mainly triacylglycerols and phospholipids.

Epidemiological studies linked high consumption of oily fish with a reduced risk of breast, prostate and colon cancer [6,7,8] in relation to the content of ω-3 PUFA. Indeed, an increased intake of ω-3 PUFAs—unlike ω-6 PUFAs, which are known to promote cancer—has well-documented anti-carcinogenic effects in vivo and in vitro [2,8,9]. These effects were reviewed by Newell et al. (2017) and more recently by Brown et al. (2020) [6,10]. For example, *in vivo*, DHA limits the progression of cancer in mouse models of breast cancer by reducing the formation of bone metastases [11] and reducing tumor growth in mouse models of ovarian cancer [12]. *In vitro*, DHA triggers the death of cultured cancer cells mainly through apoptosis [13], and can also induce cell cycle arrest [10], which is the case for prostate cancer cells [14]. In addition, DHA was shown to be an efficient adjuvant for anticancer drugs, enhancing the therapeutic effects of doxorubicin [15,16] or gemcitabine [17].

There are many DHA-rich dietary supplements that aim to compensate for a lack of this fatty acid in the diet. In these products, DHA is either esterified to triacylglycerols (TAG), which are the main lipids naturally present in fish oil, or in the form of free DHA or ethyl esters (EE), the latter two forms being obtained by chemical or enzymatic modification of fish oil TAG [18]. DHA can also be found esterified to phospholipids (PLs), such as in krill oil-derived supplements. According to Burri et al. (2012) [19], the predominant PL in marine sources such as tuna, salmon, mackerel and steelhead is phosphatidylcholine (PC), while lysophosphatidylcholine (LPC) is found in smaller amounts. Other dietary lipids with high DHA content are present in food as monoacylglycerols (MAG) and diacylglycerols (DAG) [20,21].

Special attention was paid to how the lipidic carrier in which DHA is supplied affects its bioavailability [21,22]. It is now recognized that the bioavailability of DHA in EE form is significantly lower than in TAG form [23]. In addition, the position of DHA in the TAG plays a major role, with the *sn*-2 position being preferred for a high bioavailability. Indeed, in this case, the DHA remains attached to the MAG generated by the action of pancreatic lipase and is then readily absorbed by passive diffusion [24]. In fish oil, DHA is mainly linked to the *sn*-2 position in TAG, and this is the reason why its bioavailability is higher than that of marine mammal oils, where it is linked to the *sn*-1 and *sn*-3 positions [25,26].

Concerning the DHA bound to various forms of acylglycerols, the absorption of DHA was significantly higher than free DHA, in the order MAG-DHA > DAG-DHA > TAG-DHA > free DHA [20,27]. Indeed, MAGs do not require hydrolysis by the pancreatic lipase to be absorbed [28]. Regarding the PL forms, DHA is better absorbed when provided in the form of PLs rather than in free or TAG forms [20,29]. Recently, particular interest was paid towards lysophosphatidylcholine as a carrier for DHA supply (LPC-DHA), as it was found to be more potent at enriching the brain and eyes in DHA compared to free DHA, PC-DHA (*sn*-1,2) or TAG-DHA (*sn*-1,2,3) [22,30].

In addition to the issue of DHA absorption, the influence of the carrier used to deliver DHA in preventing or treating cancer only recently began to be investigated. In this context, it was shown that, compared to free DHA, PC-DHA is more efficient for growth inhibition in colon cancer cell lines [31], DAG-DHA induces cytotoxicity with higher potency in human prostate carcinoma cells [32] and MAG-DHA is more effective in reducing the viability of various colorectal and breast cancer cell lines [33,34,35]. Regarding LPC-DHA, the only study related to cancer therapy demonstrated its ability to suppress angiogenesis in rat aortic and human umbilical vein endothelial cells in vitro [36], but no study focused on its effect on the cancer cells themselves.

**Figure 1:**
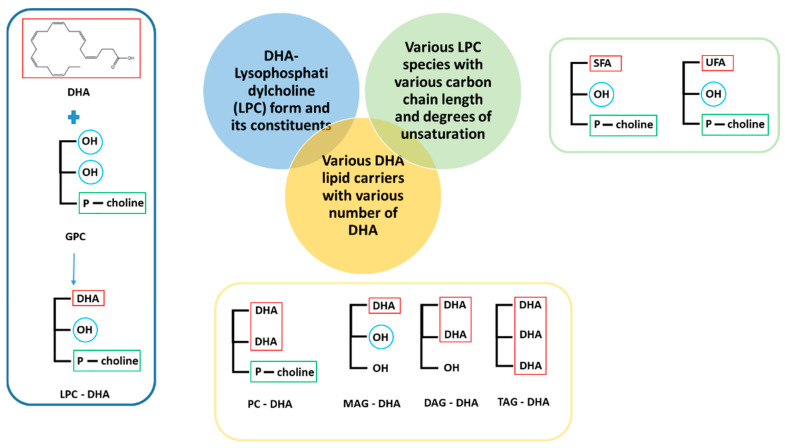
Structure of the various molecules tested in this work. SFA: saturated fatty acid, UFA: unsaturated fatty acid, PC: phosphatidylcholine, LPC: lysophosphatidylcholine, MAG: monoacylglycerol, DAG: diacylglycerol, TAG: triacylglycerol.

In this work, we performed the first systematic comparison of the effect of free DHA and DHA bound to five different lipid carriers (Figure 1) on the viability of a human cancer cell line *in vitro*. The study was performed on a breast cancer cell line because it is currently one of the most common cancers in women worldwide and it is now well known that it could be reduced by adequate dietary intake of DHA according to studies in animal models using free DHA [11,37]. Moreover, with the exception of MAG-DHA [35], the supply of DHA in a lipid-bound form to reduce breast cancer cell viability was not yet explored. The triple negative MDA-MB-231 cells were chosen as the breast cancer cells because, *in vivo*, they are associated with cancer with poor prognosis due to the absence of highly effective treatment.

Among the five different carriers studied (LPC-DHA, PC-DHA, MAG-DHA, DAG-DHA and TAG-DHA), glycerophosphocholine-based DHA carriers are much more effective in reducing the viability of MDA-MB-231 cells than free DHA and acylglycerol-based carriers, showing a strong structure-function relationship. Indeed, LPC-DHA induces considerable cytotoxicity, while PC-DHA, MAG-DHA and free DHA are less toxic. DAG-DHA and TAG-DHA have very limited effects on MDA-MB-231 viability. The potential molecular mechanisms of action of LPC-DHA were then further explored by evaluating several intracellular markers reflecting membrane and DNA damage, apoptosis, autophagy and oxidative stress.

## 2. Materials and Methods

### 2.1. DHA and Other Tested Molecules

In addition to free DHA (Interchim, Montluçon, France), various lipids containing esterified DHA were tested: 1-monodocosahexaenoin (MAG-DHA); 1,2 didocosahexaenoin (DAG-DHA); tridocosahexaenoin (TAG-DHA); didocosahexaenoyl-*sn*-glycero-3-phosphocholine (PC-DHA); 1-docosahexaenoyl-2-hydroxy-*sn*-glycero-3-phosphocholine (LPC-DHA). Other LPC derivatives carrying different fatty acids were also tested: 1-octanoyl-2-hydroxy-*sn*-glycero-3-phosphocholine (LPC-C8:0); 1-myristoyl-2-hydroxy-*sn*-glycero-3-phosphocholine (LPC-C14:0); 1-stearoyl-2-hydroxy-*sn*-glycero-3-phosphocholine (LPC-C18:0); 1-oleoyl-2-hydroxy-*sn*-glycero-3-phosphocholine (LPC-C18:1); 1-linoleoyl-2-hydroxy-*sn*-glycero-3-phosphocholine (LPC-C18:2); 1-behenoyl-2-hydroxy-*sn*-glycero-3-phosphocholine (LPC-C22:0). All molecules were purchased from Larodan (Solna, Sweden) with the exception of LPC-DHA, which was from Toronto Research Chemicals (Toronto, ON, Canada). Glycerophosphocholine (GPC), from Bachem (Bubendorf, Switzerland), which constitutes the backbone of PC and LPC, was also tested as a control.

### 2.2. MDA-MB-231 Cell Culture

MDA-MB-231 human breast cancer cells were purchased from the American Type Culture Collection (Manassas, VA, USA), and were cultured in minimal essential medium (MEM) supplemented with 10% FBS, 2 mM L-glutamine, 40 U/mL penicillin and 40 µg/mL streptomycin. Cells were incubated at 37 °C and 5% CO_2_ atmosphere. All products were purchased from Dutscher (Bernolsheim, France).

### 2.3. Treatment of MDA-MB-231 Cells with DHA and Other Molecules

Cell treatments were conducted in 96-well cell culture plates (Starstedt, Germany) that were coated with collagen (10 µg/cm^2^) (Sigma, Saint-Quentin-Fallavier, France) before use. Two hundred microliters of a suspension of MDA-MB-231 cells at 50,000 cells/mL were seeded into each well and the plate was incubated for 24 h before treatments. Cells were approximately 80% confluent at the time of treatment. A 10 mM solution of each test molecule was prepared in ethanol and diluted in a 2-fold series to a concentration of 0.3125 mM. The 6 solutions obtained were then diluted 50-fold in fresh culture medium to give treatment media with final concentrations ranging from 6.25 to 200 µM and 2% ethanol. The growth medium was removed from the wells and replaced with treatment media containing increasing concentrations of test molecules. The plate was then incubated for another 24 h.

For statistical analyses, each concentration was tested in triplicate wells of a 96-well plate, and each experiment was repeated in three independent repetitions.

In parallel with the MDA-MB-231 cell line, fibroblasts (normal adult human dermal fibroblasts) were used as a control to demonstrate the absence of effect on cell viability of LPC-DHA on non-cancerous cells. In addition, the MCF-7 breast cancer cell line was also treated with LPC-DHA and the same effect on cell viability as for MDA-MB-231 cells was observed, thus confirming the effect of LPC-DHA on breast cancer cells. These latter experiments were performed by the ImPACcell platform, Rennes, France, and the results are shown as Supplementary Data (Figure S1).

### 2.4. Evaluation of Cell Viability by Neutral Red Uptake Test

The neutral red uptake (NR, 0.4% Neutral Red solution, Sigma Aldrich, Saint Quentin Fallavier, France) test was used to evaluate cellular viability after treatment and to assess the cytotoxicity of the tested molecules. After removal of the growth medium, the cells were incubated for 2 h with 100 µL of NR solution diluted 100-fold. Cells were then washed with 100 µL of a solubilization solution (1 volume of acetic acid in 99 volumes of ethanol 50%), followed by gentle shaking for 20 min. Absorbance was measured at 540 nm using a BioTek Epoch 2 microplate reader spectrophotometer system (Agilent, Santa Clara, CA, USA).

The cell viability was expressed in % viability, calculated relative to a control consisting of untreated cells grown in the same plate. The half-maximal inhibitory concentration (IC_50_) was calculated using a four-parameter non-linear regression model in Graphpad Prism 5 (GraphPad Software, Inc., La Jolla, CA, USA).

### 2.5. Evaluation of Membrane Damage by the LDH Assay

Membrane damage induced by the different DHA lipid carriers was assessed by lactate dehydrogenase (LDH) leakage into the culture medium. The presence of LDH in the extracellular medium reflects a defect of membrane permeability which may result from necrosis and/or cell lysis. The activity of LDH in the medium was determined using the Cytotoxicity Detection Kit Plus (Sigma Aldrich, Saint Quentin Fallavier, France). The absorbances at 490 nm and 600 nm were read using a BioTek Epoch 2 microplate reader spectrophotometer system. Following the manufacturer’s instructions, the cytotoxicity of the tested molecules was calculated relative to a low control (spontaneous LDH release) and a high control (maximum LDH release) according to the equation: cytotoxicity (%) = [(A_assay_ − A_low control_)/(A_high control_ − A_low control_)] × 100, where A = A_490_ − A_600_. Each molecule was tested in triplicate wells, and three independent experiments were performed.

### 2.6. Study of Cytotoxicity by High Content Analysis

To explore the mechanism by which LPC-DHA induces cell death, different intracellular markers were studied. Apoptosis was assessed by evaluating active caspase-3 levels; oxidative stress was assessed through the quantification of heme oxygenase 1 (HO-1) and superoxide dismutase 2 (SOD-2) levels; DNA double-strand damage was studied by the quantification of phosphorylated histone 2AX (γH2AX) and the ataxia telangiectasia mutated protein (ATM phospho serine 1981); autophagy was evaluated with the microtubule-associated protein 1B-light chain 3 (LC3B) marker.

After a 24 h treatment as described above, cells were fixed 10 min with 4% formaldehyde in PBS and permeabilized with 0.2% Triton X-100. Microplates were then incubated in blocking solution (PBS with 1% BSA and 0.05% Tween-20) for 60 min before addition of primary antibodies. All antibodies were prepared in a blocking solution. The following primary and secondary antibodies were purchased from Abcam (Cambridge, UK): mouse monoclonal anti γH2AX ser139 (ab26350), rabbit polyclonal anti active caspase-3 (ab13847), rabbit polyclonal anti HO-1 (ab13293), rabbit polyclonal anti LC3B, mouse monoclonal anti ATM phospho serine 1981, goat anti-mouse IgG H&L AlexaFluor 647 (ab150115), goat anti-rabbit IgG H&L AlexaFluor 488 (ab150077). The rabbit polyclonal anti-SOD-2 antibody was provided by ThermoFisher Scientific (PA5-30604, Waltham, MA, USA).

Primary antibodies were incubated overnight at 4 °C. After washing with PBS + 0.05% Tween-20, secondary antibodies (1/2000) were incubated for 45 min at room temperature. Nuclei were stained with DAPI (1 μg/mL in PBS) for 5 min for automated cell identification by high content analysis.

Microplates were scanned with an ArrayScan VTi HCS Reader (Thermo Scientific, Waltham, MA, USA) and analyzed using the Target Activation module of the BioApplication software. For each well, 10 fields (10× magnification) were scanned and analyzed for immunofluorescence quantification. All other markers were expressed as fold increase compared to control cells. Three independent experiments were performed.

### 2.7. Statistical Analysis

Data are presented as the mean of medians ± standard deviations (SD) obtained after three independent experiments performed in triplicate. Statistical significance was evaluated using an ANOVA followed by a Dunnett post hoc test using GraphPad Prism 5 (GraphPad Software, Inc., La Jolla, CA, USA). Results with *p*-values < 0.05 were considered statistically significant.

## 3. Results

### 3.1. Effect of LPC-DHA on MDA-MB-231 Human Breast Cancer Cells

#### 3.1.1. Specificity of the Effect of LPC-DHA Compared to Free DHA and Other LPC

The human MDA-MB-231 cell line was used as an in vitro model of breast cancer in order to determine the effects of LPC-DHA. In parallel, the effect of free DHA was studied in the same conditions to compare these two forms of DHA supply to cells. The effect of GPC, which constitutes the backbone of LPC, was also tested as a control.

**Figure 2:**
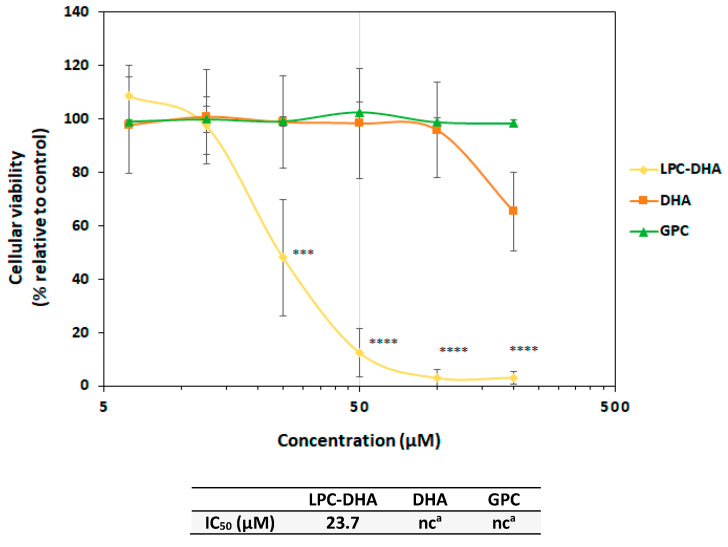
Concentration–effect relationship of LPC-DHA, DHA and GPC on MDA-MB-231 cell viability. Cells were treated with increasing concentrations (6.25, 12.5, 25, 50, 100 and 200 µM) of LPC-DHA, DHA and GPC for 24 h. Cell viability was tested with the Neutral Red Uptake test. Data are presented as mean ± SD of 3 independent experiments performed in triplicate. Statistical significance relative to the control was determined using an ANOVA with a Dunnett post hoc test. *** *p* < 0.005, **** *p* < 0.001. IC_50_ values were calculated using a four-parameter non-linear regression model in Graphpad Prism 5. In absence or too low cell viability decrease, the IC_50_ could not be calculated (^a^: nc = not calculable).

As shown in Figure 2, LPC-DHA significantly reduced the viability of MDA-MB-231 cells in vitro at concentrations higher than 25 µM (*p* < 0.005) (IC_50_ = 23.7 µM). At the highest concentrations tested (100 and 200 µM), an almost 100% loss of viability was observed. In comparison, DHA showed a much weaker effect since no effect was observed with 6.25 to 100 µM of DHA. A slight reduction in viability of 30% was observed at 200 µM. Treatment of MDA-MB-231 cells with GPC had no effect on cell viability, and the percentage of viable cells remained close to 100% at all concentrations tested. Interestingly, while LPC-DHA had a similar effect on MCF-7 cell viability, no reduction in cell counts was observed in NHDF-Ad fibroblast cells following a 24 h treatment (Figure S1).

To assess the specificity of LPC-DHA among the LPC family, MDA-MB-231 cells were treated with various commercially available LPCs carrying saturated fatty acids with different chain lengths (LPC-C8:0, LPC-C14:0, LPC-C18:0 and LPC-C22:0) or unsaturated fatty acids with different degrees of unsaturation (LPC-C18:1, LPC-C18:2 and LPC-DHA(C22:6)).

**Figure 3:**
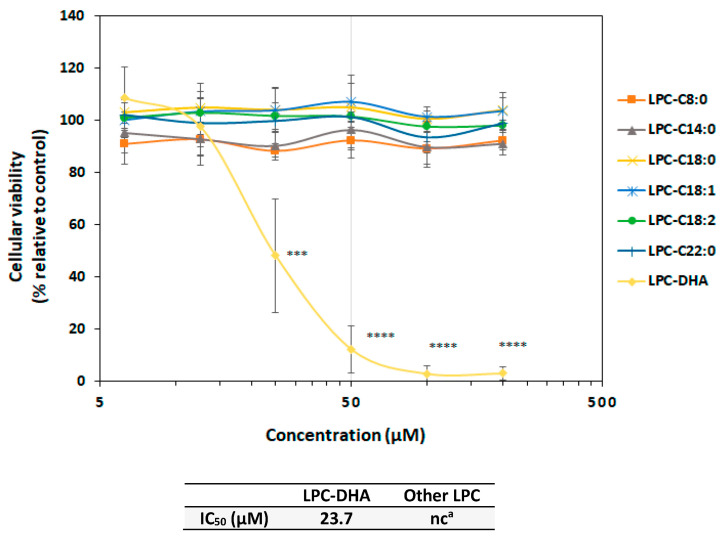
Concentration–effect relationship of various LPCs species on MDA-MB-231 cell viability. Cells were treated with increasing concentrations (6.25, 12.5, 25, 50, 100 and 200 µM) of various LPC species for 24 h. Cell viability was tested with the Neutral Red Uptake test. Data are presented as mean ± SD of 3 independent experiments performed in triplicate. Statistical significance relative to the control was determined using an ANOVA with a Dunnett post hoc test. *** *p* < 0.005, **** *p* < 0.001. IC_50_ values were calculated using a four-parameter non-linear regression model in Graphpad Prism 5. In absence or too low cell viability decrease, the IC_50_ could not be calculated (^a^: nc = not calculable).

The carbon chain length and the degree of unsaturation of the different LPC species had no influence on MDA-MB-231 cell viability as shown in Figure 3. It can, therefore, be concluded that the drastic effect of LPC-DHA on MDA-MB-231 cells was due to its unique chemical structure, assembling a phosphocholine backbone and a DHA moiety.

#### 3.1.2. Specificity of the Effect of LPC-DHA Compared to Other DHA Lipid Carriers

To further investigate the effect of LPC-DHA on MDA-MB-231 cell viability, four other lipids carrying either one, two or three DHA moieties were tested in the same conditions, namely monodocosahexaenoin (MAG-DHA), di- and tri-docosahexaenoin (DAG-DHA and TAG-DHA) and didocosahexaenoyl-phosphocholine (PC-DHA).

**Figure 4:**
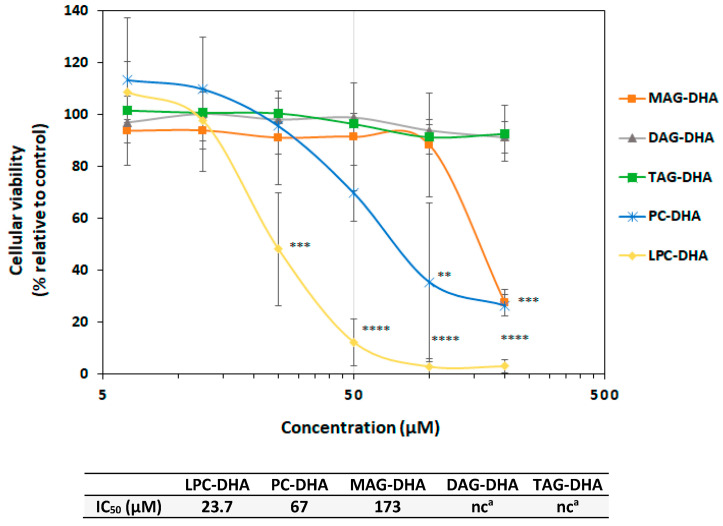
Concentration–effect relationship of lipid species containing one to three DHA chains on MDA-MB-231 cell viability. Cells were treated with increasing concentrations (6.25, 12.5, 25, 50, 100 and 200 µM) of various DHA-containing lipid species for 24 h. Cell viability was tested with the Neutral Red Uptake test. Data are presented as mean ± SD of 3 independent experiments performed in triplicate. Statistical significance relative to the control was determined using an ANOVA with a Dunnett post hoc test. ** *p* < 0.01, *** *p* < 0.005, **** *p* < 0.001. IC_50_ values were calculated using a four-parameter non-linear regression model in Graphpad Prism 5. In absence or too low cell viability decrease, the IC_50_ could not be calculated (^a^: nc = not calculable).

MAG-DHA and PC-DHA exerted significant cytotoxic effects in MDA-MB-231 cells in our conditions (Figure 4), although these species were less potent than LPC-DHA. For both compounds, the cell viability decreased by 70% at the highest concentration tested (200 µM) in comparison to untreated cells (*p* = 0.001). However, at lower concentrations, the behavior of the cells towards these two compounds was different. Indeed, cells were sensitive to PC-DHA from 50 µM, whereas for MAG-DHA, the cytotoxicity only arose at 200 µM. This difference was apparent from the IC_50_ values, with an IC_50_ for PC-DHA 2.6 times lower than that for MAG-DHA (67 µM and 173 µM, respectively). Interestingly, the acylglycerol carriers carrying two (DAG-DHA) and three DHA chains (TAG-DHA) exerted no effect on MDA-MB-231 cells, with the percentage of viable cells remaining close to 100% at all tested concentrations.

### 3.2. Investigation of Cell Death Mechanism Induced by LPC-DHA in MDA-MB-231 Cells

#### 3.2.1. LPC-DHA Did Not Induce Apoptotic or Autophagic Signaling and Did Not Provoke DNA Damage Response in MDA-MB-231 Cells

**Figure 5:**
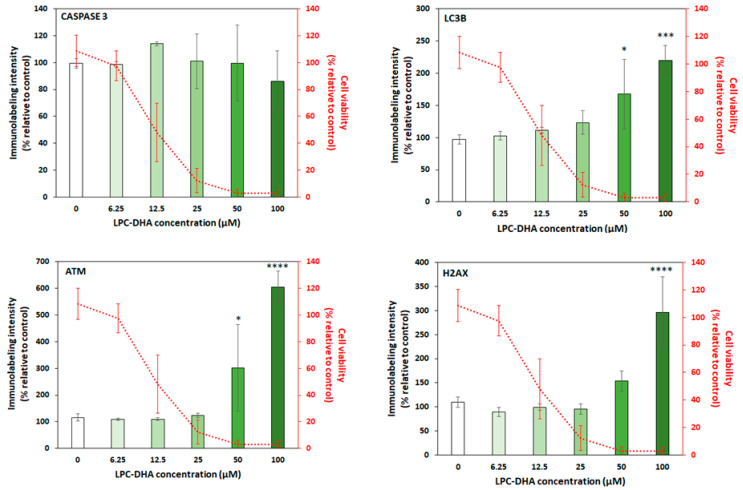
The intensity of activation of apoptosis (active caspase-3), autophagy (LC3B) and DNA damage (ATM and γH2AX) by LPC-DHA in MDA-MB-231 cells. Cells were treated with increasing concentrations (6.25, 12.5, 25, 50, 100 and 200 µM) of LPC-DHA for 24 h. Results for 200 µM were excluded from the figures as explained in the text. Data are presented as mean ± SD of 3 independent experiments performed in triplicate. Statistical significance relative to the control was determined using an ANOVA with a Dunnett post hoc test. * *p* < 0.05, *** *p* < 0.005, **** *p* < 0.001. The cell viability curve is repeated from Figure 4.

**Figure 6:**
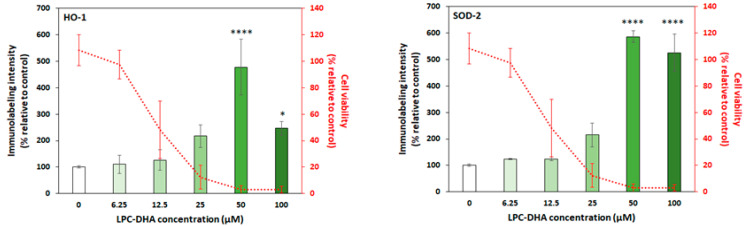
The intensity of activation of the oxidative stress (HO-1 and SOD-2 markers) by LPC-DHA in MDA-MB-231 cells. Cells were treated with increasing concentrations (6.25, 12.5, 25, 50, 100 and 200 µM) of LPC-DHA for 24 h. Results for 200 µM were excluded from the figures as explained in the text. Data are presented as mean ± SD of 3 independent experiments performed in triplicate. Statistical significance relative to the control was determined using an ANOVA with a Dunnett post hoc test. * *p* < 0.05, **** *p* < 0.001. The cell viability curve is repeated from Figure 4.

To investigate the mechanisms by which LPC-DHA was able to reduce the viability of MDA-MB-231 cells, markers of apoptosis (active caspase-3), autophagy (LC3B) and DNA damage (ATM phospho S1981 and γH2AX) were quantified (Figure 5 and Figure 6). For treatments at the 200 µM concentration, many wells did not give a labeling value, most likely due to the very low cell density resulting from the high level of cell death under these conditions. Labeling results at this concentration were, therefore, excluded from the figures. Immunolabeling intensities are expressed relative to the untreated control cells.

No change in the intensity of active caspase-3 in MDA-MB-231 cells was observed after 24 h exposure to increasing concentrations of LPC-DHA, indicating that LPC-DHA did not initiate apoptosis in our conditions (Figure 5). For the LC3B marker, an increase in intensity was observed at concentrations higher than 25 µM LPC-DHA, suggesting that autophagy could be induced by LPC-DHA in MDA-MB-231 cells.

DNA damage was studied by Ataxia telangiectasia mutated (ATM phospho S1981) and γH2AX immunolabeling. No increase in ATM marker intensity was observed in MDA-MB-231 cells exposed to LPC-DHA concentrations up to 25 µM (Figure 5). For higher concentrations, the increase observed cannot be considered representative of the mechanism of action of LPC-DHA on cells since it only appears at concentrations above the IC_50_ of LPC-DHA. This increase was likely rather a consequence of the elevated cell death at 50 µM LPC-DHA and above. Regarding γH2AX labeling, the small fluctuations of the intensities between 0 and 25 µM LPC-DHA indicated that, overall, this marker, like ATM, did not increase in cells treated with LPC-DHA. In conclusion, it is very likely that LPC-DHA did not trigger cell death by means of DNA damage.

#### 3.2.2. LPC-DHA Induces Oxidative Stress and Membrane Damage in MDA-MB-231 Cells

Oxidative stress was studied through the quantification of two enzymes: heme oxygenase-1 (HO-1), which is a stress enzyme induced in response to a variety of oxidative challenges [38], and superoxide dismutase 2 (SOD-2), which is known to control and limit the harmful effects of reactive oxygen species (ROS) [39] (Figure 6).

The loss of viability of MDA-MB-231 cells is closely related to an increase in labeling intensities for both markers (2.2-fold at 25 µM LPC-DHA) (Figure 6), suggesting an activation of the oxidative stress response mechanism following LPC-DHA treatment.

**Figure 7:**
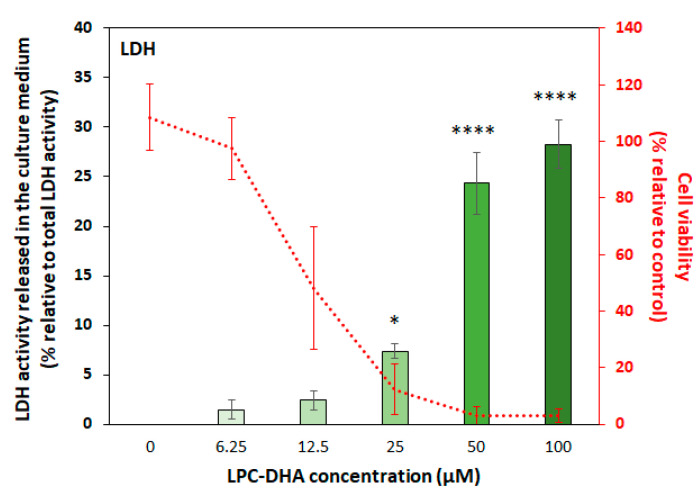
Evaluation of membrane damage in MDA-MB-231 cells upon LPC-DHA treatment. Cells were treated with increasing concentrations (6.25, 12.5, 25, 50, 100 and 200 µM) of LPC-DHA for 24 h. LDH activity released in the culture medium was measured and expressed relative to the total LDH activity in lysed cells. Results for 200 µM were excluded from the figures as explained in the text. Data are presented as mean ± SD of 3 independent experiments performed in triplicate. Statistical significance relative to the control was determined using an ANOVA with a Dunnett post hoc test. * *p* < 0.05, **** *p* < 0.001. The cell viability curve is repeated from Figure 4.

A similar correlation was also observed between cell viability and the release of lactate dehydrogenase (LDH) into the culture medium (Figure 7) which indicates that LPC-DHA induced significant membrane damage in MDA-MB-231 cells.

## 4. Discussion

In the search for bioactive compounds for healthy aging, complex lipids carrying DHA emerged as promising compounds. These DHA carriers may include TAGs, DAGs, MAGs, PLs or LPLs, and according to in vitro and preclinical studies, may have greater bioavailability [40], stronger anti-inflammatory effects [41,42] or a better capacity to improve brain function [40,43] than free DHA.

The aim of the present study was to investigate the cytotoxicity of DHA esterified on different lipid carriers on cancer cells in culture compared to free DHA. The MDA-MB-231 breast cancer cell line was chosen because the cytotoxicity of free DHA on this cell line is well known, but there is no information on the use of complex lipids for the delivery of DHA to these cells.

The lipid carriers tested in this study were synthetic lipids carrying one, two or three DHA moieties on either a glycerol backbone (MAG-DHA, DAG-DHA, TAG-DHA) or glycerophosphocholine backbone (LPC-DHA, PC-DHA).

**Figure 8:**
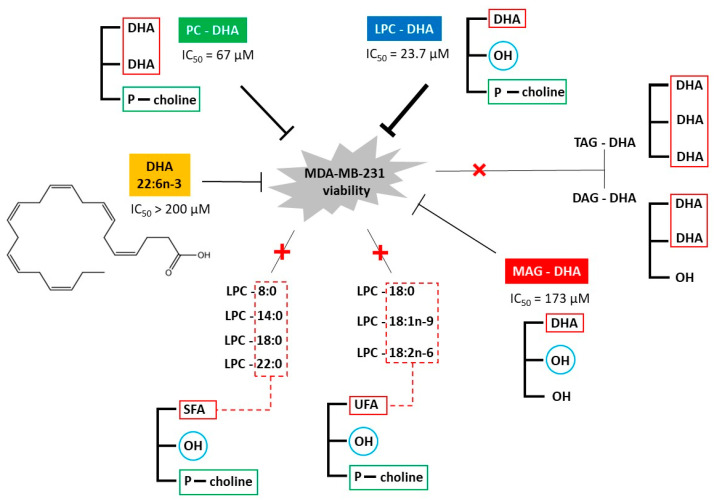
The biological effect of the different DHA lipid carriers and the various LPC species. SFA: saturated fatty acid, UFA: unsaturated fatty acid, PC: phosphatidylcholine, LPC: lysophosphatidylcholine, MAG: monoacylglycerol, DAG: diacylglycerol, TAG: triacylglycerol. The thickness of the inhibition lines (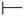) are in relation to the IC_50_ values. The red cross on the line indicates no effect of the tested molecules.

According to our findings, the glycerophosphocholine-based DHA carriers (LPC-DHA and PC-DHA) were the most effective in reducing the viability of MDA-MB-231 cells, with LPC-DHA being more effective (IC_50_ = 23.7 µM) than PC-DHA (IC_50_ = 67 µM). Both lipids were even more effective than free DHA. For glycerol-based carriers, MAG-DHA showed higher cytotoxicity (IC_50_ = 173 µM) than free DHA, but lower than LPC-DHA and PC-DHA. Finally, DAG-DHA and TAG-DHA did not show any effect on cell viability under our conditions (Figure 8). These findings showed a direct relationship between the chemical structure of the DHA carrier and the cytotoxicity.

In the literature, a concentration of 100 µM of free DHA was reported to be significantly cytotoxic to MDA-MB-231 cells [44,45], whereas in Rizzo et al., 2021 [46] and in our work, no effect was observed at concentrations below 200 µM. Explanations for these variations in results include the differences in the experimental procedures used. For instance, the DHA was added to the culture medium either solubilized in DMSO [46], associated with a high amount of albumin (ratio of albumin/DHA of 5:1) [46], which facilitates the transport across the cell membrane [47] or as an ethanolic solution (this work). Moreover, the number of cells, the cultivation time of cells prior to the treatment and the duration of the treatment vary from one study to another which results in experimental conditions that are not entirely comparable.

LPC-DHA was not extensively studied in the context of DHA delivery to the organism or supply to cells in culture, probably because it is found in low amounts in natural sources of DHA such as microalgae, fish or krill oils. However, it was commercially available since 2018 and is increasingly being studied. Studies were even carried out to describe different synthesis routes, in particular by enzymatic reaction with lipases [48,49]. To our knowledge, the only study on the effect of LPC-DHA in the context of cancer therapy focused on tumor angiogenesis and showed that LPC-DHA at 100 µM allowed an effective suppression of angiogenesis on rat main artery and human umbilical cord vein endothelial cells in vitro [36]. According to our study, LPC-DHA also exerted significant effects on the cell viability of the MCF-7 breast cancer cell line, giving rise to similar IC_50_ value. However, no cytotoxicity was observed in NHDF-Ad fibroblasts following a 24 h treatment (Figure S1). This suggests that the effect of LPC-DHA could be specific for cancer cell lines.

To further characterize the cytotoxic potential of LPC-DHA, we verified that the cytotoxicity was not solely attributable to one or specific part of the molecule (GPC and DHA) and that the whole molecule is, therefore, essential. We also showed that nine other LPCs, with fatty acids of 8–22 carbon chain length and 0–2 double bonds, did not alter cell viability, demonstrating a very strong specificity of the structure-activity relationship of LPC-DHA.

The impact of various LPCs on the viability of cultured cancer cells was described in the literature, but not for breast cancer cells or LPC-DHA. LPC-C16:0, LPC-C18:0 and LPC-C18:1 are cytotoxic in leukemia cancer cells (Jurkat T cells) at 20 µM in a specific manner, as a wide range of other LPLs, namely saturated LPC with shorter (6–14 carbon) and longer (19–24 carbon) chain, LPA, LPS, LPE and LPG, were not cytotoxic [50]. Interestingly, cytotoxicity was observed only when cells were cultured in FBS-free medium. In the presence of FBS, Jurkat T cells were protected from LPC-induced cytotoxicity in a concentration-dependent manner, with a concentration of 5% FBS giving rise to a complete absence of cytotoxicity. The inhibition of cytotoxicity was due to LPC binding to the albumin contained in FBS. If the lack of cytotoxicity of some LPCs under our conditions is due to FBS in the culture medium, then it can be concluded that LPC-DHA does not interfere with albumin in the same way as other LPCs. In another study, LPC-C18:0, at a concentration of 450 µM, was found to reduce the metastatic spread of murine melanoma B16.F10 cells [51] but lower concentrations were not tested.

The effect of MAG-DHA was studied on some cancer cell lines, including colon cancer and breast cancer [34,35], but MDA-MB-231 cells were not included in these studies. MAG-DHA is a potent cytotoxic compound towards SKBR3 and E0771 breast cancer lines [35], with an IC_50_ of 20 µM and 16 µM, respectively. Regarding DAG-DHA, it is more cytotoxic for prostate carcinoma cells than free DHA [32]. The toxicity of PC-DHA was studied only on colon [31] and lung [52] cancer cell lines. It was shown that PC-DHA decreased these cancer cell viabilities more potently than DHA. Overall, these data indicate that the quantitative effect of various DHA lipid carriers on cancer cell viability in vitro is highly dependent on the cancer cell type and the experimental conditions.

The results obtained with the three glycerol-based carriers (MAG-DHA, DAG-DHA and TAG-DHA) showed that increasing the number of DHA chains on the molecule resulted in a loss of cytotoxicity. A similar phenomenon occurred with the glycerophosphocholine-based carriers as well, as PC-DHA was less cytotoxic than LPC-DHA. In addition, the greater cytotoxicity of the LPC-DHA compared to the MAG-DHA is indicative of the importance of the head group. Thus, it appears that the mode of DHA delivery to cells, rather than the amount of DHA delivered, may be essential for high cytotoxicity.

LPC-DHA is a DHA carrier known to facilitate DHA uptake into tissues more efficiently than PC-DHA [22,30]. We, therefore, suggest that its higher cytotoxicity towards MDA-MB-231 comes from its higher ability in enriching the cell with DHA. Similarly, the cytotoxic effect of MAG-DHA could be explained by the better absorption of DHA with this carrier than with DAG-DHA and TAG-DHA [20].

In an attempt to investigate the intracellular mechanisms involved in LPC-DHA cytotoxicity, various biological markers were measured in treated cells. Surprisingly, LPC-DHA does not induce apoptosis (active caspase 3 levels), although this is the main described pathway by which free DHA leads to MDA-MB-231 cell death [10]. LPC-DHA does not induce autophagy (LC3B marker) either, nor DNA damage (ATM and H2AX markers).

Significant increases in oxidative stress markers (HO-1 and SOD-2) were observed when cells were treated with a concentration of LPC-DHA above 25 µM.

Membrane damage is the mechanism most strongly correlated with the loss of MDA-MB-231 cell viability due to LPC-DHA treatment. It is well known that extracellular lysophospholipids, due to their amphiphilic nature, can translocate into the cell membrane lipid bilayer. Their incorporation into the membrane induces changes in the molecular organization of the membrane and ion permeability, ultimately leading to cell death through disruption of cell membranes [53,54]. However, in our case, we would have observed cell cytotoxicity not only with LPC-DHA but also with other LPCs if the observed membrane damage was the result of an LPC-mediated increase in membrane permeability.

Thus, in this study, LPC-DHA may preferentially increase ROS in or near the plasma membrane lipid rafts, and trigger lipid peroxidation due to the high level of DHA, which lead to the activation of the antioxidant defense system as shown by increased expression of HO-1 and SOD-2. Indeed, the unsaturated nature of DHA makes cells more sensitive to free radicals because it makes the membrane less rigid and more vulnerable, which causes lipid peroxidation leading to membrane damage [55].

To summarize, we demonstrated that LPC-DHA, PC-DHA, MAG-DHA and free DHA exerted cytotoxic effects in cultured MDA-MB-231 breast cancer cells, with LPC-DHA being the most potent followed by PC-DHA, MAG-DHA and free DHA (Figure 8). Under the same conditions, DAG-DHA and TAG-DHA did not have a cytotoxic effect. These results, therefore, showed a strong correlation between the molecular structure of DHA-carrying lipids and their ability to reduce MDA-MB-231 cell viability *in vitro*. The wide variety of DHA carriers tested in this work made it possible to show that (i) DHA carriers based on glycerophosphocholine were more potent than those based on acylglycerol and (ii) carriers with one DHA chain were more potent than those with two or three DHA chains. In comparison to the literature, our work, therefore, extends the important question of the structure of DHA carriers into the context of cancer cell viability *in vitro*, which was not the subject of research to date. Moreover, it was shown that the potent cytotoxic effect of LPC-DHA was not associated with apoptosis, as described in the literature for free DHA, but rather with oxidative stress, which could be due to lipid peroxidation, which in turn induced membrane cell damage.

This first study showing a cytotoxic effect of LPC-DHA on MDA-MB-231 cells in vitro suggests that this compound may have advantages in the context of breast cancer. However, due to the heterogeneity of breast cancer cell types, the results need to be validated in other cell lines, in particular the MCF7 line, and the mechanism of action needs to be further elucidated. In addition, this study used the sn1 isomer of LPC-DHA, which is not the main isomer naturally found in the body. While some studies, in the context of brain enrichment [22] and retinal enrichment [30], showed similar effects with both isomers, the comparison of the two isomers should also be addressed in breast cancer cells.

## Data Availability

Not applicable.

## Supplementary Materials

The following supporting information can be downloaded at: https://www.mdpi.com/article/10.3390/nu15092137/s1, Figure S1: Concentration-effect relationship of LPC-DHA on breast cancer cell lines (MDA-MB-231 and MCF-7) and a non-cancer cell line (NHDF-Ad; Normal Human Dermal Fibroblasts, Adult).
