# Supplementary material for: Lysophosphatidylcholine-DHA Specifically Induces Cytotoxic Effects of the MDA-MB-231 Human Breast Cancer Cell Line In Vitro—Comparative Effects with Other Lipids Containing DHA
Source: Nutrients. 2023 Apr 29;15(9):2137. doi: 10.3390/nu15092137 (PMC10180881; doi:10.3390/nu15092137)
Supplement: Supplementary file 1 [file nutrients-15-02137-s001.zip › nutrients-2263910-supplementary.pdf]

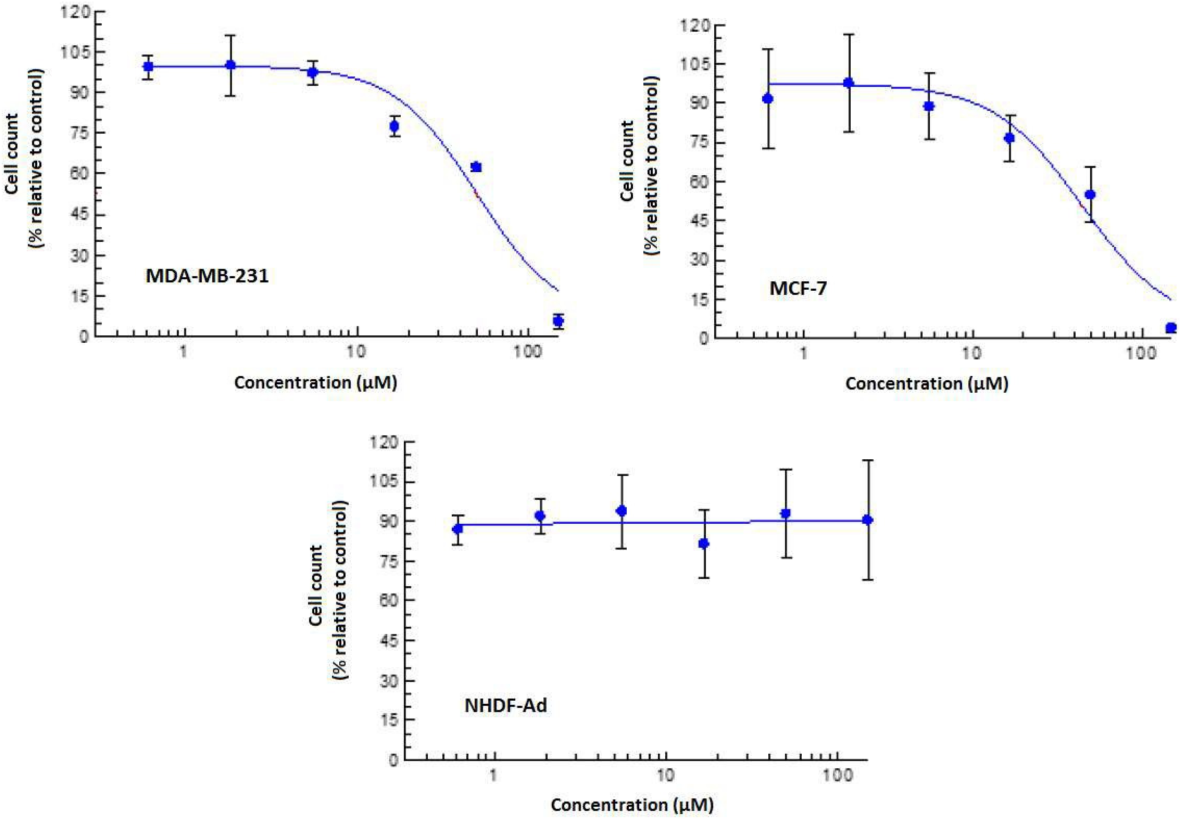

Figure S1. Concentration-effect relationship of LPC- DHA on breast cancer cell lines (MDA-MB-231 and MCF-7) and a non-cancer cell line (NHDF-Ad ; Normal Human Dermal Fibroblasts, Adult). Cells were treated with increasing concentrations (0.6, 1.8, 5.5, 16.7, 50 and 150 µM) of LPC-DHA for 24 hrs. Cell viability was determined by counting the number of nuclei that have incorporated DAPI using an ArrayScan IV HCS reader. Relative cell counts are expressed relative to an untreated control. Data are presented as mean ± SD of 3 independent experiments
